# Celiac disease and liver

**Published:** 2010-12-01

**Authors:** Pekka Collin

**Affiliations:** 1 Department of Gastroenterology and Alimentary Tract Surgery, Tampere University Hospital, Tampere, Finland

Dear Editor,

Leonardi and La Rosa [1] investigated the occurrence of celiac disease in patients with hepatitis B by screening their sera with anti-endomysial and anti-tissue transglutaminase antibodies-both of which are sensitive serological tests for celiac disease. The authors found no one with celiac disease in 60 patients who had contracted hepatitis B infection in childhood. As they admitted, the power of the study was too low to make any definitive conclusions.Celiac disease is defined as "a permanent intolerance to ingested gluten, the structural protein in wheat rye and barley." The ingestion of gluten results in small bowel mucosal inflammation, crypt hyperplasia and villous atrophy. Typically, patients suffer from diarrhea, abdominal complaints, malaise, and malabsorption with resultant iron deficiency anemia. The histpathology and symptoms resolve on gluten-free diet.Of note, the condition is often asymptomatic. Extraintestinal manifestations or associated autoimmune conditions may be the sole presentations of celiac disease. Here, I disagree with Leonardi and La Rosa [1] who stated that associated autoimmune diseases are usually found in celiac patients with severe complications: existing autoimmune disorders (e.g., autoimmune hepatitis) may be the only disease manifestation in an otherwise asymptomatic celiac patient [2].Isolated hypertransaminasemia is common in celiac disease, and conversely, unexplained hypertransaminasemia may be a diagnostic clue in celiac disease. Celiac disease has been reported to occur in patients with primary biliary cirrhosis, autoimmune hepatitis and primary sclerosing cholangitis [3]. Untreated celiac disease may exacerbate the associated liver disease, and gluten-free dietary treatment in such cases, may result in a dramatic improvement, even in advanced liver disease [4] .As to the viral hepatitis, there are more studies on the occurrence of celiac disease in hepatitis C than in hepatitis B. Two large studies comprising 624 [5] and 878 [6] patients with hepatitis C found no increased frequency of celiac disease. The positive associations are mainly based on case reports.When considering the reports, some facts must be taken into account. Firstly, celiac disease affects more than 1% of people in many countries, which means that the disease associations may be fortuitous. For instance, if we make an estimate that 1% of the population in Italy is infected by hepatitis B virus, it means that 6000 people with hepatitis B will have (mostly undetected) celiac disease; and there would be even more cases with hepatitis C and celiac disease. Secondly, symptoms of celiac disease are variable and subtle. There are case reports where celiac disease has been activated after the commencement of interferon therapy [6]. This is yet to be proved, however. Patients treated with interferon are usually kept on careful surveillance, and side-effects of interferon may mimic symptoms of celiac disease (i.e., anemia, depression, and fatigue). It is thus possible that serologic screening will be carried out more easily in patients who have received medical therapy for viral hepatitis than in other patients suffering from similar symptoms. Nevertheless, the development of celiac disease should be kept in mind during interferon therapy, as the drug may activate latent autoimmune diseases.Evidence suggests that patients with celiac disease have a lower response rate to hepatitis B vaccination than healthy subjects. This failure to respond may increase the risk of hepatitis B in celiac disease [7].To conclude, at the moment, there is no convincing evidence that patients with viral hepatitis carry an increased risk of celiac disease. The results of Leonardi and La Rosa [1] support this conclusion. However, the protean and subtle clinical manifestations of celiac disease should be remembered when treating patients with hepatitis B or C.

## References

[1] Leonardi S, La Rosa M (2010). Are Hepatitis B Virus and Celiac Disease Linked?. Hepat Mon.

[2] Collin P, Kaukinen K, Valimaki M, Salmi J (2002). Endocrinological disorders and celiac disease. Endocr Rev.

[3] Rubio-Tapia A, Murray JA (2007). The liver in celiac disease. Hepatology.

[4] Kaukinen K, Halme L, Collin P, Färkkilä M, Mäki M, Vehmanen P, Partanen J, Höckerstedt K (2002). Celiac disease in patients with severe liver disease: gluten-free diet may reverse hepatic failure. Gastroenterology.

[5] Thevenot T, Denis J, Jouannaud V, Monnet E, Renou C, Labadie H, Abdelli N, Nguyen-Khac E, Dumouchel P, Bresson-Hadni S, Chousterman M, DI Martino V, Cadranel JF (2007). Coeliac disease in chronic hepatitis C: a French multicentre prospective study. Aliment Pharmacol Ther.

[6] Hernandez L, Johnson TC, Naiyer AJ, Kryszak D, Ciaccio EJ, Min A, Bodenheimer HC Jr, Brown RS Jr, Fasano A, Green PH (2008). Chronic hepatitis C virus and celiac disease, is there an association?. Dig Dis Sci.

[7] Leonardi S, Spina M, Spicuzza L, Rotolo N, La Rosa M (2009). Hepatitis B vaccination failure in celiac disease: is there a need to reassess current immunization strategies?. Vaccine.

